# VTd-PACE and VTd-PACE-like regimens are effective salvage therapies in difficult-to-treat relapsed/refractory multiple myeloma: a single-center experience

**DOI:** 10.1007/s00277-022-05027-y

**Published:** 2022-11-16

**Authors:** Susanne Ghandili, Dzenefa Alihodzic, Christian Wiessner, Carsten Bokemeyer, Katja Weisel, Lisa B. Leypoldt

**Affiliations:** 1 Department of Oncology, Hematology and Bone Marrow Transplantation With Section Pneumology, University Cancer Center Hamburg, Martinistraße 52, 20246 Hamburg, Germany; 2 Hospital Pharmacy, University Medical Center Hamburg-Eppendorf, Martinistraße 52, 20246 Hamburg, Germany; 3 Institute for Medical Biometry and Epidemiology, University Medical Center Hamburg‐Eppendorf, Martinistraße 52, 20246 Hamburg, Germany

**Keywords:** Multiple myeloma, Relapse, VTd-PACE, Salvage therapy

## Abstract

Although treatment options for multiple myeloma (MM) are rapidly evolving, there still remain difficult-to-treat situations, especially in relapsed and/or refractory (r/r) disease. When modern therapies are exhausted, or emergency treatment is needed for high tumor burden, classic chemotherapy combination regimens like the VTd-PACE regimen and its modifications (PACE-M) may also be beneficial as bridging to subsequent treatment options. This single-center retrospective analysis aimed to investigate the outcome of VTd-PACE and PACE-M salvage therapy in 31 heavily pretreated r/r MM patients. The primary objective was the overall response rate (ORR). Secondary objectives were median progression-free survival (mPFS), median overall survival (mOS), safety, and renal response. Median age was 59 years (range 39–75), and 71% of patients were male. R-ISS stratification showed high-risk MM in 48%. The median number of prior therapies was 3, with 23 patients being triple- and 12 penta-refractory (74% and 39%). ORR was 71%, including 23% of patients achieving a very good partial response. Median duration of follow-up was 15 months (range 0–29 months). mPFS and mOS were 3 months (95% CI 0.27–5.74) and 11 months (95% CI 3.66–18.35), respectively. In 26 patients (83.9%), at least one subsequent treatment (stem cell transplant or BCMA-directed) was administered. Renal function significantly improved after VTd-PACE or PACE-M treatment (*p* = 0.032). Non-hematological adverse events ≥ grade 3 were predominantly infections. VTd-PACE and PACE-M are effective salvage therapies in difficult-to-treat situations in heavily pre-treated r/r MM, including patients with impaired renal function. VTd-PACE and PACE-M can be successfully used as bridging therapy for subsequent treatment.

**Supplementary Information:**

The online version contains supplementary material available at 10.1007/s00277-022-05027-y.

## Introduction

Multiple myeloma (MM) is the second most common hematological malignancy worldwide [1]. With the development of novel drugs, MM has become a malignant disease in which, during the last two decades, a smooth transition from conventional chemotherapy to immunotherapy and targeted therapy has been achieved. Although MM remains incurable, the use of proteasome inhibitors, immunomodulating drugs, and, most recently, monoclonal antibodies has significantly prolonged progression-free survival (PFS) and overall survival [2]. However, although treatment options rapidly evolve in MM, there still remain difficult-to-treat situations, especially when novel treatments are exhausted. For penta-refractory MM, defined as patients who are refractory to at least two proteasome inhibitors, two immunomodulating agents, and a monoclonal CD38-directed antibody, median overall survival (mOS) was reported to be no longer than 5.6 months [3]. Overall response rate (ORR) is low, with a recent real-world study showing an ORR of only 20% to the subsequent applied therapy for triple-class exposed patients that were almost all refractory to their last treatment line [3, 4]. With the failure of modern treatment options, classic chemotherapy combinations like the VTd-PACE regimen, a combination of bortezomib, thalidomide, dexamethasone, cisplatin, doxorubicin, cyclophosphamide, and etoposide, and its modifications (PACE-M) are used as salvage therapy. However, systematic data of those regimens in the era of novel agents are rare [5–13]. Initially, the efficacy of PACE-based regimes was not only used for patients with relapsed or refractory (r/r) MM, but also investigated in patients with newly diagnosed MM. In 2003, Lee et al. investigated the feasibility of two cycles of a combination of 4 days of oral dexamethasone, daily thalidomide, and 4 days of a continuous infusion of cisplatin, doxorubicin, cyclophosphamide, and etoposide (DT-PACE) in a total of 236 patients with r/r MM. By integrating and modifying the posttransplant consolidation regimen of the TOTAL THERAPY-2 trial by adding daily thalidomide, Lee et al. demonstrated an ORR (defined as partial response (PR) or better) in 32% after two cycles of DT-PACE [14, 15]. With the addition of the first-in-class proteasome inhibitor bortezomib to the DT-PACE protocol, the TOTAL THERAPY-3 phase-II trial investigated the efficacy of two cycles VTd-PACE as induction treatment, followed by a melphalan-based tandem transplant regimen and a consolidation with two additional cycles of VTd-PACE in 303 patients with newly diagnosed MM. This was followed by 3-year maintenance of monthly cycles of VTd in the first year and thalidomide and dexamethasone alone in the remaining 2 years. After 2 years, 83% of patients achieved a near-complete response and a 2-year estimated event-free survival and OS of 84% and 86%, respectively, after 20 months of follow-up [16]. We here report a single-center retrospective analysis to investigate the feasibility of VTd-PACE and PACE-M as salvage therapy in patients with r/r MM, who have exhausted novel treatment options.

## Methods

### Study design and patients

In this single-center retrospective study, we included patients aged 18 years and older, with a confirmed diagnosis of r/r MM who received at least one cycle of VTd-PACE or any modified version of the TOTAL THERAPY-3 trial VTd-PACE. VTd-PACE and PACE-M were chosen as salvage therapy for patients with triple- or penta-refractory or relapsed MM and an immediate need of response due to ongoing myeloma-related end-organ damage (e.g., acute kidney injury, bone marrow failure). All patients included in this analysis were diagnosed with MM according to the 2014 updated diagnostic criteria of the international myeloma working group (IMWG) [17], or were diagnosed with primary plasma cell leukemia. Patients with a diagnosis of systemic light-chain amyloidosis were excluded.

Whenever the required data was available, disease staging (ISS and revised ISS stage) was performed according to IMWG criteria [17], and extramedullary MM was defined according to Bhutani et al. [18]. Prior to the first cycle of VTd-PACE or PACE-M, treatment lines were counted as recommended by Rajkumar et al. [19], triple- and penta-refractory disease were defined similarly to Gandhi et al. [3], and the presence of any of del(17p), 1q amplification with > 3 copies, t(4;14), t(14;16), or t(14;20) was considered as high-risk cytogenetic feature [20–22]. Remission status for MM was evaluated according to the 2016 updated IMWG consensus criteria for response and minimal residual disease assessment in MM [23].

VTd-PACE regimen was applied according to Barlogie et al., with bortezomib being applied subcutaneously. In detail, a 28-day cycle of VTd-PACE consisted of bortezomib at a dose of 1 mg/m^2^ body surface area (BSA) on days 1, 4, 8, and 11, dexamethasone 40 mg given orally on days 1, 2, 3, and 4, thalidomide 200 mg taken orally once a day throughout the cycle and continuous intravenous (IV) infusions from day 1–4, which consisted of cisplatin 10 mg/m^2^ BSA/day, doxorubicin 10 mg/m^2^ BSA/day, cyclophosphamide 400 mg/m^2^ BSA/day, and etoposide 40 mg/m^2^ BSA/day [16]. Dose modifications on an individual basis (e.g., due to renal failure or polyneuropathy) were performed according to standard practice and are discussed below. Supportive treatments (anti-infective prophylaxis, thrombosis prophylaxis, growth factor support, red blood cell, and platelet transfusions) were administered according to local standards. Response assessments took place after the first and, if applicable, each subsequent cycle of VTd-PACE or PACE-M. The treating physician decided about additional cycles up to a maximum of four cycles. The overall response rate (ORR) was defined as achieving a PR, or better, and clinical benefit rate (CBR) was defined as achieving a minor response (MR) or better. For the assessment of patient comorbidities, Charlson Comorbidity Index (CCI) was used [24]. All toxicities were assessed using the Common Terminology Criteria for Adverse Events (CTCAE) version 5 [25].

All included patients were treated at the Department of Oncology and Hematology at the University Medical Center Hamburg-Eppendorf, Germany, and were identified by a systematic search of the pharmacy chemotherapy dataset for a combination of the above-mentioned drugs, namely a combination of cisplatin, doxorubicin, cyclophosphamide, and etoposide, that were produced between January 2015 and March 2022. The clinical data regarding treatment and disease characterization were collected from the patient’s electronic medical records. The data cutoff was on April 5, 2022.

The retrospective data collection was performed in accordance with local legal requirements. It was reviewed and approved by the Ethics Committee of the Medical Council of Hamburg (vote number 2022–100,775-BO-ff), and the ethics committee waived informed consent since only pseudonymous data were analyzed and published.

### Study endpoints

The primary aim of this study was to evaluate the ORR of VTd-PACE and PACE-M in settings of r/r MM and difficult-to-treat situations. Secondary aims included PFS and OS data, and detailed analyses regarding defined subgroups (e.g., high-risk cytogenetic aberration, renal insufficiency). Moreover, we aimed to compare the toxicity and adverse events of the applied regimes.

### Statistical analysis

All statistical analyses were performed, and figures were created using the Statistical Package for Social Sciences statistical software, version 27.0, R and RStudio, version 1.4.1717 with the package survminer and Microsoft Excel for Mac, version 16.38. Continuous variables are presented as median with range. Categorical variables are expressed as numbers and percentages (%) and compared by Fisher’s exact test. Group differences in continuous variables were tested by a *t* test. The time-to-event analyses were performed using the Kaplan–Meier estimator; groups were compared using the log-rank test. Univariate analysis was accomplished by applying the Cox regression model to identify a possible relationship between variables of interest (high-risk cytogenetic aberrations, penta-refractory disease, extramedullary manifestation, lactate dehydrogenase above the upper limit of normal, and leukocytopenia or thrombocytopenia prior to a treatment with VTd-PACE or PACE-M and shortened OS. *P* values < 0.05 were considered as statistically significant. The reported *p* values are two-tailed.

## Results

### Patients’ characteristics

Thirty-one patients who received VTd-PACE or a modified PACE-regimen for r/r MM between January 2015 and March 2022 were identified by a systematic search of the pharmacy chemotherapy dataset and included in the analysis. Patients’ demographics and characteristics are presented in Table 1. The median age was 59 years (range 39–75), and the majority of patients were male (71%). Median CCI was 2. R-ISS stratification showed high-risk MM with R-ISS stage III in 10 of 21 evaluable patients (48%, R-ISS was not assessable in ten patients) with high-risk cytogenetic aberrations in 25 out of 29 evaluable patients (86%). The most common cytogenetic aberration was amplification of 1q (> 3 copies) in 17 patients (59%), followed by del(17p) in 14 patients (48%), t(4;14) in six (21%), and t(14;16) in three patients (10%). A total of 13 patients (45%) showed more than one high-risk aberration, and 15 patients (48%) had elevated levels of lactate dehydrogenase above the upper limit of normal as an additional high-risk feature. Central nervous system involvement was known in one patient (3%), and extramedullary disease was seen in 19 patients (61%). Overall, all patients had r/r disease (100%). Of those, 23 were triple- and 12 penta-refractory (74% and 39%, respectively). The median number of prior treatment lines was three (range 1–10). Twenty-four patients (77%) underwent prior autologous stem cell transplantation (up to three times), of whom two (6%) additionally received an allogeneic transplantation. The median number of VTd-PACE or PACE-M treatment cycles applied in this case series was two (range 1–4). Regarding treatment modifications for PACE-M refer to supplementary table 1.

**Table 1:** Patients’ characteristics

Total number of patients, *n*	31
Age at starting PACE-M, years, median (range)	59 (39–75)
Male sex, *n* (%)	22 (71)
CCI median (range)	2 (2–6)
Isotype, *n* (%)	
IgG	14 (45)
IgA	6 (19)
Light chain	10 (32)
Other	1 (3)
CNS involvement, *n* (%)	1 (3)
R-ISS stages, *n* (%)	
I	1 (3)
II	10 (32)
III	10 (32)
Not evaluable	10 (32)
High risk cytogenetics ^a^, *n* (%)	25 (86)
del(17p)	14 (48)
t(4;14)	6 (21)
t(14;16)	3 (10)
Amplification 1q (> 3 copies)	17 (59)
> 1 high-risk aberration	13 (45)
Extramedullary disease (EMD), *n* (%)	19 (61)
Extraosseous EMD	9 (47)
Bone-related EMD	12 (63)
Lactate dehydrogenase > upper limit of normal, *n* (%)	15 (48)
Triple refractory, *n* (%)	23 (74)
Penta-refractory, *n* (%)	12 (39)
Prior lines of therapy, median (range)	3 (1–10)
Prior stem cell transplant, *n* (%)	
Autologous PBSCT	24 (77)
Allogeneic PBSCT	2 (6)
Number of patients treated each year, *n*	
2015	0
2016	0
2017	0
2018	0
2019	6
2020	10
2021	13
2022	2

*CNS* central nervous system, *CCI* Charlson Comorbidity Index, *PBSCT* peripheral blood stem cell transplant

^a^Evaluable in 29 of 31 patients

### Treatment response and survival analysis

**Fig. 1:**
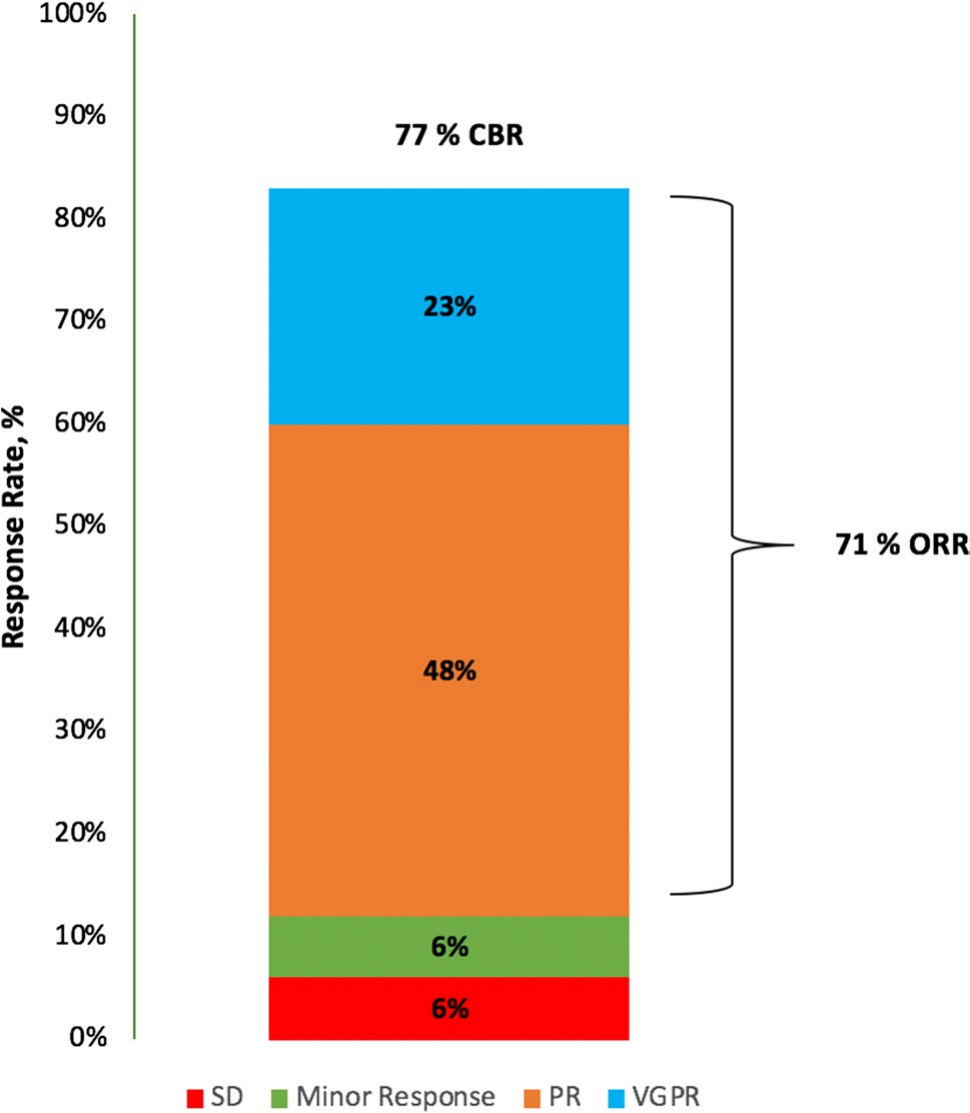
Overall response rate (ORR) and clinical benefit rate (CBR) of VTd-PACE and PACE-M

**Fig. 2:**
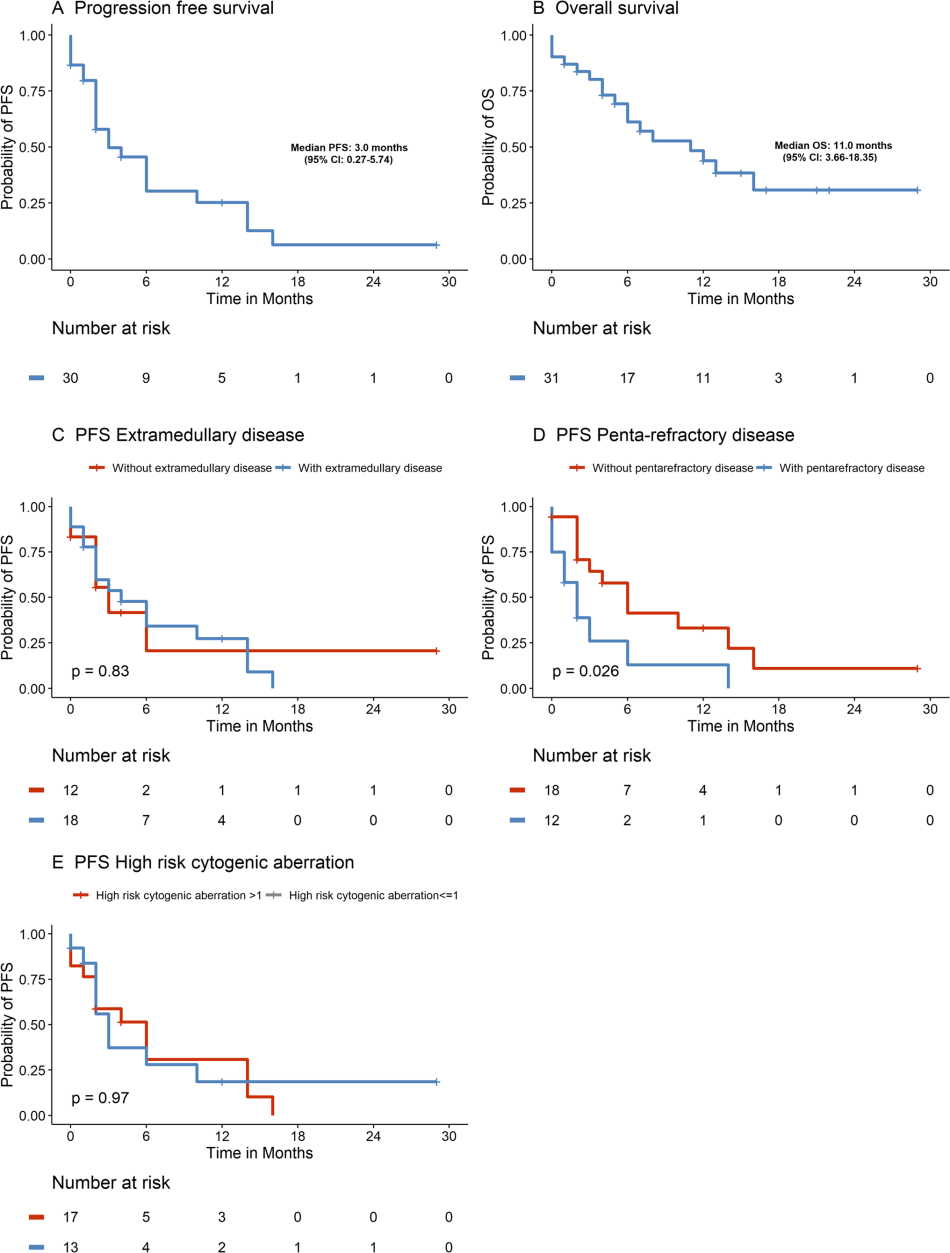
Kaplan–Meier plots for median progression-free survival (mPFS) (**A**) and median overall survival (mOS) (**B**) in patients treated with VTd-PACE or PACE-M. Comparisons of Kaplan–Meier plots for median progression-free survival (mPFS) between patients depending in number of high-risk cytogenetic aberrations (**E**), extramedullary manifestation (**C**), and penta-refractory disease (**D**)

The median duration of follow-up was 15 months (range 0–29 months). ORR to VTd-PACE or PACE-M was 71%. A total of 22 patients (71%) achieved PR or better including seven patients (23%) who achieved VGPR. Stable disease (SD) was achieved in two patients (6%) (refer to Fig. 1). Median PFS (mPFS) was 3 months (95% CI 0.27–5.74) (Fig. 2A). There was no significant difference regarding PFS in patients with > 1 high risk cytogenetic aberrations compared to those with ≤ 1 high risk cytogenetic aberration (mPFS 3 months (95% CI 1.5–4.5) vs. 6 months (95% CI 2.2–9.8), *p* = 0.97) (Fig. 2C). Furthermore, PFS did not differ significantly between patients with and without extramedullary manifestation (mPFS 4 months (95% CI 0.3–7.7) vs. 3 months (95% CI 0.7–5.3), *p* = 0.83) (Fig. 2D). In penta-refractory disease, mPFS was significantly decreased compared to patients with less than penta-refractory disease (mPFS 2 months (95% CI 0.5–3.5) vs. 6 months (95% CI 2.9–9.1), *p* = 0.026) (Fig. 2E). Median OS was 11 months (95% CI 3.66–18.35) (Fig. 2B).

A total of 26 patients (84%) received at least one subsequent therapy line. Subsequent therapies consisted of high-dose melphalan-based autologous stem cell transplantation, allogeneic stem cell transplantation, and BCMA-directed treatment approaches (including treatment with belantamab mafodotin, BCMAxCD3 directed bispecific antibodies, and BCMA-directed CAR-T-cell therapy) in 26%, 3%, and 29%, respectively. Other subsequent therapies included predominantly pomalidomide-based combinations in 26%, two patients received treatment with the GPRC5DxCD3 bispecific antibody talquetamab as part of clinical trials, and CAR-T-cells are currently being manufactured for one patient. A total of 21 patients (68%) achieved a platelet count of > 75.000/m^3^. Cytopenia and renal failure are common complications preventing patients from being included in clinical trials. In this cohort, severe cytopenia (defined as grade 3 or 4 leukopenia or thrombocytopenia) due to MM-related bone marrow insufficiency was prevalent in 17 patients (55%) already at the start of treatment. Renal function was significantly impaired with a GFR < 60 ml/min in eleven patients (35%) but improved significantly after the end of treatment with VTd-PACE or PACE-M (mean GFR: 66.7 vs. 83.6 ml/min, *p* = 0.032). Infection-related and bleeding-related causes of non-relapse-related death occurred in three and one patient, respectively. Of those, one death is possibly related to VTd-PACE treatment (severe course of COVID-19). Moreover, the cause of death is unknown in five patients.

### Analysis of predicting factors

Next, we investigated the impact of several patient and disease-specific characteristics regarding the prediction of survival in patients treated with VTd-PACE or PACE-M. In univariate Cox regression analysis neither high-risk cytogenetic aberrations, penta-refractory disease, extramedullary manifestation, lactate dehydrogenase above the upper limit of normal, nor leukocytopenia prior to a treatment with VTd-PACE or PACE-M was associated with shortened OS. Thrombocytopenia prior to a treatment with VTd-PACE or PACE-M was significantly associated with shortened OS (*p* = 0.046).

### Adverse events

Grade 3 or 4 cytopenia was observed in all 31 patients including 45 episodes of grade 3 or 4 neutropenia in 31 patients, 42 episodes of grade 3 or 4 lymphocytopenia in 30 patients, and 46 episodes of grade 3 or 4 thrombocytopenia in 30 patients (supplementary Fig. 1). The median duration of neutropenia and thrombocytopenia were 6 and 14 days, respectively. In total, seven bleeding events (grade 3 or 4) related to thrombocytopenia occurred including two intracerebral bleedings and two gastrointestinal bleedings. Non-hematological adverse events ≥ grade 3 occurred in 26 patients, which were predominantly infections including 22 episodes of neutropenic fever, three events of COVID19, one herpes simplex-virus pneumonia, one esophageal candidiasis, one respiratory syncytial-virus infection, and one rotavirus infection. Furthermore, two episodes of grade 3 emesis and one episode of grade 3 enoral mucositis occurred. Pre-existing renal failure required dose modifications of VTd-PACE or PACE-M in 32.3% of patients but allowed a safe administration.

## Discussion

Even though treatment options are rapidly evolving in MM, there still remain difficult-to-treat situations, particularly in heavily pretreated r/r MM. With the failure of modern treatment options, and most recent developments such as bispecific antibodies and CAR-T-cell therapies not yet widely available, classic chemotherapy combination regimens like the VTd-PACE regimen and its modifications may be beneficial in these situations. By investigating the clinical course of 31 heavily pretreated patients with r/r MM receiving VTd-PACE and PACE-M as salvage therapy, we observed an ORR of 71% which is in line with previously reported ORR of 67.7%, 68.4%, 73%, 75%, and 77% [8, 10, 11, 26, 27] and is significantly higher than the lately described ORR of 20% to the next applied therapy for triple-class exposed patients [4]. Usually, complete responses only account for a minor part of the ORR reported for VTd-PACE or PACE-M with 0–14% [7–11, 13, 27–29]. Thus, we do not consider the lack of complete responses in our study population as unusual, as bone marrow evaluations for response documentation were not performed routinely in clinical practice, but see our results in line with previously reported data of most retrospective studies [5, 7, 9, 12, 13, 28].

Moreover, we observed a mPFS of 3 months which is consistent with the results of Lakshman and colleagues who investigated the efficacy of VTd-PACE and PACE-like regimen in r/r MM in the largest retrospective study population to date, with a mPFS of 3.1 months [13], while some studies reported slightly higher mPFS rates of 4.5, 5.0, and 5.5 months [7–9]. However, we deem the here reported mPFS remarkable, since all patients had been exposed to novel treatments and were in urgent need of efficacious treatment options at the time of VTd-PACE or PACE-M application. With a mPFS of three months, VTd-PACE or PACE-M can compare to the PFS seen in r/r MM clinical trials with belantamab mafodotin or selinexor with mPFS of 2.8 months and 3.7 months, respectively, in which smaller percentages of patients showed high-risk features [30, 31].

In this study, we observed a mOS of 11 months which is paralleled by the majority of previously reported mOS rates of 8–14 months in patients with heavily pretreated r/r MM [7–9, 13, 29]. However, in studies including mainly r/r MM-patients who received less than three treatment lines prior to VTd-PACE or in newly diagnosed MM, mOS of more than 28 and even 42.5 months were reported [10, 27]. In our study, only one patient died due to a non-relapse related cause of death which is possibly related to VTd-PACE treatment (severe course of COVID-19). However, as VTd-PACE and PACE-like regimen result in longer lasting episodes of severe cytopenia, adequate supportive treatment including transfusions and early detection and treatment of infections is key.

Remarkably, 32% of patients responding to salvage chemotherapy with VTd-PACE or PACE-M in our cohort were able to receive subsequent next treatments like BCMA-directed antibody–drug conjugate, BCMA- or GPRC5D-targeting T-cell-engaging bispecific antibodies, and BCMA-targeting CAR-T-cell therapy mostly within clinical trials. This is an important fact supporting the use of VTd-PACE or PACE-M as salvage treatments to allow r/r MM patients to be bridged to novel clinical trial options and to be stabilized or recover regarding bone marrow function and renal impairment.

However, in univariate Cox regression analyses, factors typically associated with a negative prognostic outcome, such as high-risk cytogenetic aberrations, penta-refractory disease, extramedullary manifestation, lactate dehydrogenase above the upper limit of normal, and leukocytopenia prior to a treatment with VTd-PACE or PACE-M, were not associated with shortened OS. This may partly be reflected by a selection and confounding bias of the population since all patients showed clinical high-risk features in just different combinations. Regarding cytogenetics, our results are paralleled by previous studies that neither observed a significant association of high-risk cytogenetic aberrations on OS [8, 9, 13]. However, regarding the impact of extramedullary disease on OS, our results are consistent with other studies investigating VTd-PACE or PACE-M in r/r MM [8, 10, 13]. In our cohort thrombocytopenia prior to a treatment with VTd-PACE or PACE-M was the only factor significantly associated with a shortened OS in univariate Cox regression analysis.

Due to the relatively small cohort size and the retrospective study design, there is a risk of potential selection bias as discussed above, of residual confounding, and a low power in this analysis. Therefore, our findings remain to be confirmed in larger trials. In addition, due to the real-world clinical routine character of this trial, there was no minimal residual disease assessment and partially also lack of frequent bone marrow biopsies to further assess responses.

Despite the short duration of response, VTd-PACE and PACE-M can be effective salvage therapies in difficult-to-treat r/r MM patients and may be useful as bridging therapy to other effective treatments, e.g., clinical trial options, BCMA-directed therapies, CAR-T-cell therapy, treatment with bispecific antibodies, or melphalan-based high dose therapy with autologous stem cell transplantation. For these situations, VTd-PACE and PACE-M may still remain in the armamentarium of MM treatment options even in the era of specialized and targeted treatments.

## Supplementary Information

Below is the link to the electronic supplementary material.

Supplementary file1 (PPTX 45.5 KB)

Supplementary file2 (DOCX 16.2 KB)

## References

[1] Sung H, Ferlay J, Siegel RL (2021). Global cancer statistics 2020: GLOBOCAN estimates of incidence and mortality worldwide for 36 cancers in 185 countries. CA Cancer J Clin.

[2] Ghandili S, Weisel KC, Bokemeyer C, Leypoldt LB (2021). Current treatment approaches to newly diagnosed multiple myeloma. Oncol Res Treat.

[3] Gandhi UH, Cornell RF, Lakshman A (2019). Outcomes of patients with multiple myeloma refractory to CD38-targeted monoclonal antibody therapy. Leukemia.

[4] Mateos M-V, Weisel K, Stefano VD (2021). LocoMMotion: a prospective, non-interventional, multinational study of real-life current standards of care in patients with relapsed/refractory multiple myeloma (RRMM) receiving ≥3 prior lines of therapy. J Clin Oncol.

[5] Srikanth M, Davies FE, Wu P (2008). Survival and outcome of blastoid variant myeloma following treatment with the novel thalidomide containing regime DT-PACE. Eur J Haematol.

[6] Magen-Nativ H, Ram R, Yeshurun M (2010). Total therapy-based treatment for multiple myeloma—a single center experience. Ann Hematol.

[7] Gerrie AS, Mikhael JR, Cheng L (2013). D(T)PACE as salvage therapy for aggressive or refractory multiple myeloma. Br J Haematol.

[8] Griffin PT, Ho VQ, Fulp W (2015). A comparison of salvage infusional chemotherapy regimens for recurrent/refractory multiple myeloma. Cancer.

[9] Huynh T, Corre E, Lemonnier MP (2021). Role of D(T)PACE-based regimens as treatment of multiple myeloma with extramedullary relapse or refractory disease. Leuk Lymphoma.

[10] Ainley L, Chavda SJ, Counsell N (2021). DT-PACE/ESHAP chemotherapy regimens as salvage therapy for multiple myeloma prior to autologous stem cell transplantation. Br J Haematol.

[11] Abdallah AO, Sigle M, Mohyuddin GR (2021). Outcomes of VD-PACE with immunomodulatory agent as a salvage therapy for relapsed/refractory multiple myeloma. Clin Lymphoma Myeloma Leuk.

[12] Cowan AJ, Green DJ, Karami M (2020). KRD-PACE mobilization for multiple myeloma patients with significant residual disease before autologous stem-cell transplantation. Clin Lymphoma Myeloma Leuk.

[13] Lakshman A, Singh PP, Rajkumar SV (2018). Efficacy of VDT PACE-like regimens in treatment of relapsed/refractory multiple myeloma. Am J Hematol.

[14] Barlogie B, Tricot G, Anaissie E (2006). Thalidomide and hematopoietic-cell transplantation for multiple myeloma. N Engl J Med.

[15] Lee CK, Barlogie B, Munshi N (2003). DTPACE: an effective, novel combination chemotherapy with thalidomide for previously treated patients with myeloma. J Clin Oncol.

[16] Barlogie B, Anaissie E, van Rhee F (2007). Incorporating bortezomib into upfront treatment for multiple myeloma: early results of total therapy 3. Br J Haematol.

[17] Rajkumar SV, Dimopoulos MA, Palumbo A (2014). International Myeloma Working Group updated criteria for the diagnosis of multiple myeloma. Lancet Oncol.

[18] Bhutani M, Foureau DM, Atrash S (2020). Extramedullary multiple myeloma. Leukemia.

[19] Rajkumar SV, Richardson P, San Miguel JF (2015). Guidelines for determination of the number of prior lines of therapy in multiple myeloma. Blood.

[20] Palumbo A, Avet-Loiseau H, Oliva S (2015). Revised international staging system for multiple myeloma: a report from International Myeloma Working Group. J Clin Oncol.

[21] Fonseca R, Bergsagel PL, Drach J (2009). International Myeloma Working Group molecular classification of multiple myeloma: spotlight review. Leukemia.

[22] Avet-Loiseau H, Attal M, Campion L (2012). Long-term analysis of the IFM 99 trials for myeloma: cytogenetic abnormalities [t(4;14), del(17p), 1q gains] play a major role in defining long-term survival. J Clin Oncol.

[23] Kumar S, Paiva B, Anderson KC (2016). International Myeloma Working Group consensus criteria for response and minimal residual disease assessment in multiple myeloma. Lancet Oncol.

[24] Charlson ME, Pompei P, Ales KL, MacKenzie CR (1987). A new method of classifying prognostic comorbidity in longitudinal studies: development and validation. J Chronic Dis.

[25] Common Terminology Criteria for Adverse Events (CTCAE) Version 5.0. (2018) In: https://ctep.cancer.gov/protocoldevelopment/electronic_applications/ctc.htm#ctc_50. Accessed 14 June 2022

[26] Alsouqi A, Khan M, Dhakal B (2021). KD-PACE salvage therapy for aggressive relapsed refractory multiple myeloma, plasma cell leukemia and extramedullary myeloma. Clin Lymphoma Myeloma Leuk.

[27] Muchtar E, Ram R, Raanani P (2014). First line and salvage therapy with total therapy 3-based treatment for multiple myeloma—an extended single center experience. Leuk Res.

[28] Buda G, Orciuolo E, Galimberti S (2013). VDTPACE as salvage therapy for heavily pretreated MM patients. Blood.

[29] Beyer K, Rosner S, Woo KM (2014). Analysis of VDT-PACE utilization in multiple myeloma patients treated at MSKCC for relapsed disease or cytoreduction and stem cell mobilization after initial induction therapy. Blood.

[30] Lonial S, Lee HC, Badros A (2021). Longer term outcomes with single-agent belantamab mafodotin in patients with relapsed or refractory multiple myeloma: 13-month follow-up from the pivotal DREAMM-2 study. Cancer.

[31] Chari A, Vogl DT, Jagannath S (2019). Selinexor for refractory multiple myeloma. Reply N Engl J Med.

