# Supplementary material for: VTd-PACE and VTd-PACE-like regimens are effective salvage therapies in difficult-to-treat relapsed/refractory multiple myeloma: a single-center experience
Source: Ann Hematol. 2022 Nov 16;102(1):117–24. doi: 10.1007/s00277-022-05027-y (PMC9667441; doi:10.1007/s00277-022-05027-y)
Supplement: Supplementary file 1 — Supplementary file1 (PPTX 45.5 KB) [file 277_2022_5027_MOESM1_ESM.pptx]

## Slide 1
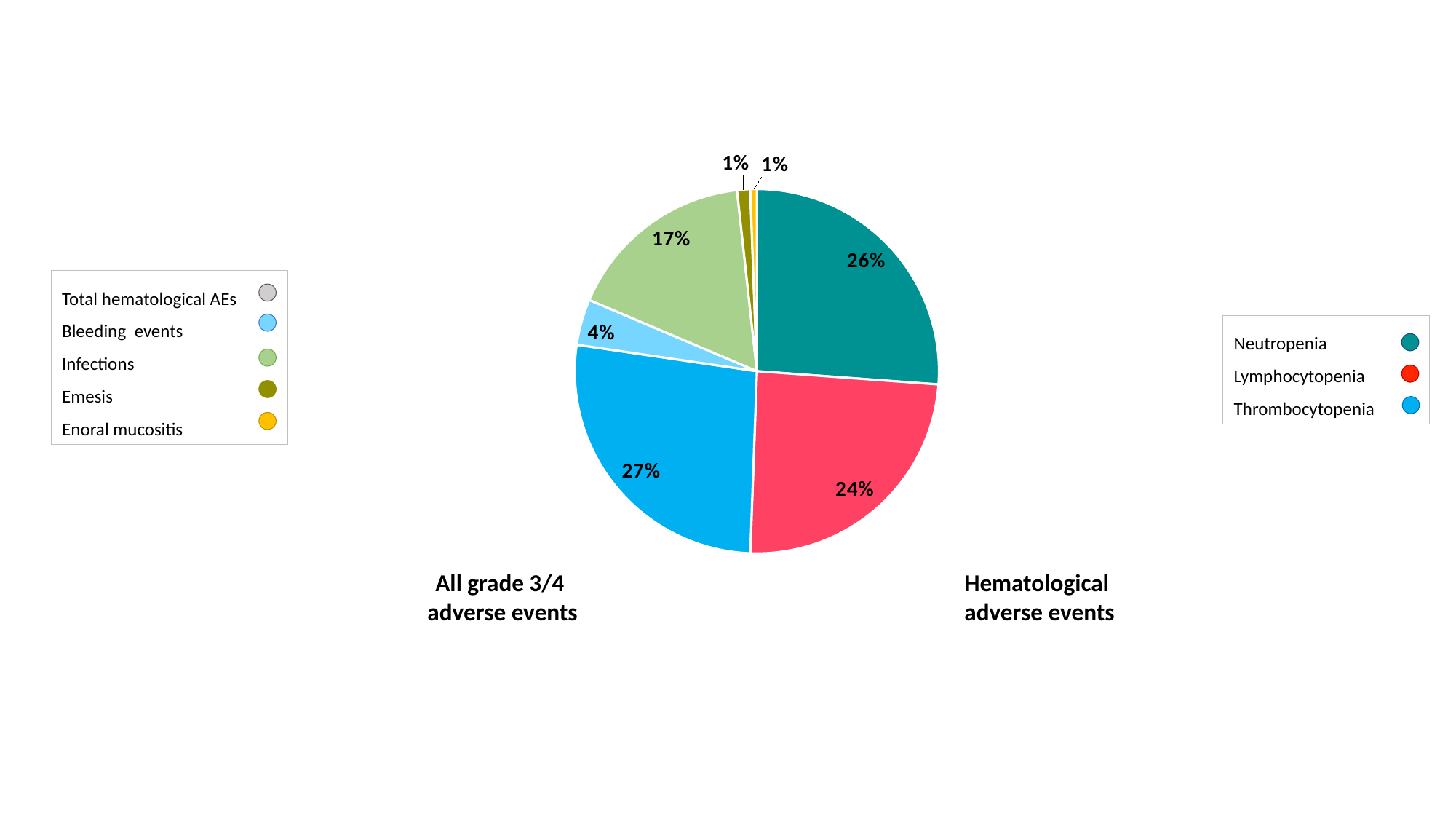

[unsupported chart]
Total hematological AEs
Bleeding events
Infections
Emesis
Enoral mucositis
Neutropenia
Lymphocytopenia
Thrombocytopenia
Hematological
adverse events
All grade 3/4
adverse events
