# Supplementary material for: VTd-PACE and VTd-PACE-like regimens are effective salvage therapies in difficult-to-treat relapsed/refractory multiple myeloma: a single-center experience
Source: Ann Hematol. 2022 Nov 16;102(1):117–24. doi: 10.1007/s00277-022-05027-y (PMC9667441; doi:10.1007/s00277-022-05027-y)
Supplement: Supplementary file 2 — Supplementary file2 (DOCX 16.2 KB) [file 277_2022_5027_MOESM2_ESM.docx]

Supplementary figure **1**: Overview of the distribution of grade 3/4 adverse events related to VTd-PACE or PACE-M.

Supplementary table **1**: Overview of treatment regimens defining PACE-M, including modification rational, response to PACE-M and PACE-M-related adverse events for each patient.

| **PACE-M treatment regimens** | **Description of VTd-PACE modification** | **Rational of VTd-PACE modification** | **Number of patients treated, n** | **Response to PACE-M:**  **Best Response/PFS (in months)/OS (in months)** | **Grade 3/4 adverse events** |
| --- | --- | --- | --- | --- | --- |
| Dara-VTd-PACE | Addition of daratumumab to VTd-PACE | Both patients did not receive any CD38-directed treatment. For this reason, we chose to add daratumumab to increase the VTd-PACE efficacy. | 2 | Patient 1: VGPR/6/11  Patient 2: PR/NA/4 | Patient 1: Neutropenia, lymphocytopenia, and thrombocytopenia  Patient 2: Neutropenia, lymphocytopenia, and thrombocytopenia |
| KdT-PACE | Use of a second-generation PI: carfilzomib instead of bortezomib | Carfilzomib was preferred over bortezomib due to extramedullary disease progression during prior treatment with bortezomib-containing regimen | 1 | Patient 3: VGPR/NA/12 | Patient 3: Neutropenia, neutropenic fever, lymphocytopenia, and thrombocytopenia |
| VdP-PACE | Use of a third-generation IMiD: pomalidomide instead of thalidomide | Pomalidomide was preferred over thalidomide due to progression during prior first-line treatment consisting of isatuximab, carfilzomib, lenalidomide and dexamethasone (as part of a clinical trial) to overcome lenalidomide refractoriness | 1 | Patient 4: Refractory/0/1 | Patient 4: Neutropenia, neutropenic fever, lymphocytopenia, and thrombocytopenia |
| KRd-PACE flowed by tandem melphalan-based high dose chemotherapy and maintenance with carfilzomib and lenalidomide | Use of a second-generation PI: carfilzomib instead of bortezomib and use of a second-generation IMiD: lenalidomide instead of thalidomide | Carfilzomib was preferred over bortezomib and lenalidomide over thalidomide due to progression during/refractoriness to prior first-line treatment consisting of daratumumab, bortezomib, thalidomide and dexamethasone to overcome VTd-refractoriness and second due to progression of extramedullary disease | 1 | Patient 5: PR/14/21 | Patient 5: Neutropenia, neutropenic fever, lymphocytopenia, and thrombocytopenia, enorale mucositis |

PI: Proteasome inhibitor, IMiD: Immunomodulatory drug, PFS: Progression-free survival, OS: Overall survival, NA: Not applicable, VGPR: Very good partial response, PR: Partial response

Supplementary table **2**: Overview of publications regarding outcome of VTd-PACE and PACE-M treatment regimens

| **Authors and year** | **Regimen** | **Trial phase** | **Patient number (n)** | **Overall response rate** | **Complete response rate** | **Median progression-free survival (PFS) (months)** | **Median overall survival (OS) (months)** | **Comments** |
| --- | --- | --- | --- | --- | --- | --- | --- | --- |
| Huynh T et al., 2021 [9] | D(T)-PACE-based | monocentric retrospective | 43 | 58% | 14% | 5.0 | 9.0 | Cytogenetics had no impact on response rate, overall survival and progression-free survival |
| Alsouqi A et al., 2021 [26] | KD-PACE | 2-center retrospective | 52 | 77% | 12% | 4.6 | 11.2 | Patients bridged to autologous or allogeneic hematopoietic stem cell transplant, or a clinical trial had a superior PFS and OS |
| Ainley L et al., 2021 [10] | DT-PACE/ESHAP | single-centre retrospective | 63, of those 38 with DT-PACE | 71% | 14% | 7.9 | 28.9 | Median prior lines of therapy: 1 (range 1-4) |
| Abdallah AO et al, 2021 [11] | DT-PACE | single-centre retrospective | 30 | 67.7% | 13% | 11.0 | 26.0 |  |
| Lakshman A et al., 2018 [13] | VDT PACE | single-centre retrospective | 141 | 54.4% | 0.7% | 3.1 | 8.1 |  |
| Griffin PT et al., 2015 [8] | DCEP, VTD-PACE, CVAD | single-centre retrospective | 107, of those 22 with VDT-PACE | 73% | 9% | 4.5 | 8.5 |  |
| Gerrie AS et al., 2013 [7] | D(T)-PACE | 2-center retrospective | 75 | 49% | 0% | 5.5 | 14.0 |  |
| Srikanth M et al., 2008 [5] | DT-PACE | single-centre retrospective | 26 | 59% | NA | 3.0 | 7.0 | Blastoid variant myeloma |
